# Addressing the stigma and promoting awareness: Electroconvulsive therapy in Pakistan

**DOI:** 10.1002/hsr2.2197

**Published:** 2024-06-11

**Authors:** Esha Tir Radia Ghori, Muhammad Abdul Wasay Zuberi, Tirth Dave, Varisha Fatima Shaikh

**Affiliations:** ^1^ Department of Medicine Baqai Medical University Karachi Pakistan; ^2^ Department of Medicine Dow University of Health Sciences Karachi Pakistan; ^3^ Department of Medicine Bukovinian State Medical University Chernivtsi Ukraine

Electroconvulsive therapy (ECT) is a medical treatment for psychiatric disorders in which an electric current is passed through the brain to induce a seizure for therapeutic purposes.^1^ It is one of the oldest treatments for mental disorders, being first described 85 years ago, and continues to be among psychiatry's highly effective treatment options.^2^ It is mainly used to treat and manage depression, manic episodes, catatonia, and treatment‐resistant schizophrenia.^3^ Over the past few decades, while mental health professionals have primarily relied on antipsychotic medications, evidence shows that in 20%–30% of patients, the response rate to conventional antipsychotics is poor, particularly, in people with schizophrenia.^4^

ECT is now proving vital in managing severe forms of depression and other mental illnesses. All over the world, there has been a significant increase in the use of ECT over the years, while in Pakistan, the use of ECT is still deficient as there is a stigma attached to mental health issues. ECT is a low‐risk treatment and relatively safe during pregnancy, as psychotropic drugs carry risks for pregnant patients and their unborn babies.^5^ It is the most potent treatment in psychiatry and is more effective than pharmacotherapy for severe unipolar depression, according to a meta‐analysis published in Lancet.^6^ ECT is frequently used as an effective life‐saving treatment for acutely suicidal patients.^7^ Research has shown multiple pathways by which ECT most likely exerts its mode of action including multiple neuroendocrine and immune changes, which involve hyperconnectivity, neuroinflammation reduction, hypothalamus–pituitary–thyroid axis changes, among other effects.^8^ Saeed et al. conducted research at a tertiary care hospital in Sindh, Pakistan, which showed that both right unilateral ECT and bilateral ECT were highly effective in treating depression.^9^ Research has also shown that people prescribed ECT have shorter hospital stays than those who used antipsychotic drugs.^10^

Administration of unmodified ECT has been a common practice in Pakistan. According to Minhas et al.'s analysis of ECT practice in Rawalpindi, Pakistan, 89.9% patients were administered unmodified ECT.^11^ Due to stigma and inadequate knowledge regarding ECT, it remains underprescribed, particularly, in Pakistan.^12^ It has multiple controversies attached to adverse public perceptions about giving people an electrical stimulus, safety concerns, disputes over effectiveness, and potential consequences, such as memory loss.^13^ Due to a lack of research on mental health and psychiatric disorders in Pakistan, ECT has not been studied, resulting in a lack of knowledge about ECT which is not then optimally utilized for the benefit of the patients. There are not enough trained doctors and psychiatrists, in Pakistan. Also, due to various social and cultural factors, along with a low literacy rate, mental health problems are generally stigmatized.^14^

There is considerable stigma attached to ECT in Pakistan, as people believe that the induction of seizures Germany: a web‐based survey. Psychiatry may result in brain damage due to the electric current. Some people consider the procedure barbaric, and promote the negative claims about ECT, for example, that it alters the structure of the brain.^15^ In a study conducted at a university hospital in Karachi, Pakistan, out of 4013 patients admitted, only 136 received ECT over 13 years.^12^ This emphasizes the reluctance of people to use ECT. According to a cross‐sectional survey conducted by Waqas et al., in which 527 people responded out of 620, 13.7% people believed that spiritual leaders are best able to treat mental illnesses, while 32.1% people considered that it is black magic that causes mental disorders.^16^ The results of their cross‐sectional survey not just highlight that there is still a lack of awareness regarding mental health issues in Pakistan but also support the idea that more research, and education is required to improve the general understanding of mental disorders, so that we can become more accepting of the treatments like ECT.

While ECT can be highly effective for people with treatment‐resistant schizophrenia and severe forms of depression, it is also important to investigate the adverse effects associated with ECT. Postictal confusion, characterized by restlessness, anxiety, and disorientation occurs in about 8%–20% of people receiving ECT.^17^ Transient side effects of ECT include headaches (usually mild and self‐limiting), myalgia, neurologic deficits like aphasia (lasting a few hours after ECT), and anterograde and retrograde (most persistent) amnesia. Soon after ECT, most patients have impaired memory for events that occurred close in time to the course of ECT and stretches back several months or years.^18^

It is important to research the differences in outcome between patients who receive ECT and those who are treated with psychotropic medication. It is noteworthy that even after several decades of healthcare practitioners trying to improve awareness regarding mental health in Asia, the acceptance and use of ECT is still much less compared to Western countries.^19^ This can be addressed by clinical experts highlighting the positive outcomes from ECT and by providing ECT training programs.^20^ An ECT education training program was also conducted in British Columbia, Canada, in which about 73 student nurses and 21 care aid students participated. This was followed by a questionnaire. More favorable and positive attitudes were reported toward ECT upon completion of the program as compared to attitudes before the training program.^20^ Therefore, ECT can be destigmatized by educating people on the subject and doctors can play their part in this by explaining the procedure and comparing pros and cons of antipsychotic drugs with those of ECT.

According to the World Health Organization, 24 million people in Pakistan currently need psychiatric support, and there are only 0.19 psychiatrists per 100,000 population. Keeping in mind the increasing number of patients with mental illnesses and a literacy rate of only about 62.3%,^21^ it has become even more critical to conduct awareness campaigns, and destigmatize ECT. With so little ECT being prescribed, physicians are unable to make advancements in the field and develop expertise, perpetuating the cycle. Furthermore, researchers should be encouraged to explore and investigate the adverse effects of ECT and analyze its benefits and disadvantages compared to those of psychotropic medications, so that patients are better able to make an informed decision.

## AUTHOR CONTRIBUTIONS

All authors contributed equally in drafting, revising and submitting the final version of the manuscript.

## CONFLICT OF INTEREST STATEMENT

The authors declare no conflict of interest.

## TRANSPARENCY STATEMENT

The lead author affirms that the manuscript is an honest, accurate, and transparent account of the study being reported.

## Data Availability

The data used in this study are publicly available from the National Library of Medicine https://www.ncbi.nlm.nih.gov/. All relevant data are included in the manuscript. Additional data related to this study may be requested from the corresponding author upon reasonable request.
